# Heterogeneous Chlorine Reactions on Mineral Dust During Dust Storm Events in the Coastal City of Qinhuangdao

**DOI:** 10.3390/toxics14060460

**Published:** 2026-05-25

**Authors:** Yulong Wang, Jiajia Shao, Ting Wei, Ruihe Lyu, Pengju Liu, Chen Lin, Wenhua Wang, Longyi Shao

**Affiliations:** 1 School of Resources and Civil Engineering, Northeastern University, Shenyang 110819, China; 2 School of Resources and Materials, Northeastern University at Qinhuangdao, Qinhuangdao 066004, China; 3 College of Marine Resources and Environment, Hebei Normal University of Science & Technology, Qinhuangdao 066004, China; 4 State Key Laboratory of Regional Environment and Sustainability, School of Environment, Tsinghua University, Beijing 100084, China; 5 College of Geosciences and Surveying Engineering, China University of Mining and Technology, Beijing 100083, China; shaol@cumtb.edu.cn

**Keywords:** dust storm, single particle, heterogeneous reaction, chlorine chemistry

## Abstract

Heterogeneous reactions on mineral dust surfaces during dust storm events significantly influence atmospheric chemistry by modifying aerosol composition. This study employed high-resolution scanning electron microscopy coupled with energy-dispersive X-ray spectroscopy (SEM-EDX) to investigate the morphology and elemental composition of dust particles collected during four dust events in Qinhuangdao, a coastal city in Northern China. The results revealed that clay minerals were the most abundant (53.5% ± 13.7%), followed by feldspar (12.0% ± 3.6%), quartz (11.9% ± 3.2%) and carbonate minerals (7.3% ± 4.1%). In contrast, sulfate and NaCl particles constituted only a minor fraction, representing 1.1% and 0.9% of the particles, respectively. Elemental analysis indicated that sulfur (S)-containing particles, although scarce during the initial stages, increased as the dust storms evolved, eventually accounting for about half of the particles by the end of the events. This trend suggests increasing heterogeneous processing on dust particle surfaces. Furthermore, single-particle analysis demonstrated a marked increase in chlorine (Cl)-containing particles (excluding NaCl) from 2.4% at the onset to 30.9% ± 16.5% by the end of the dust events. In contrast to studies conducted in inland cities, where Cl-containing particles are seldom observed, our findings underscore the crucial role of marine aerosols in facilitating the chlorine enrichment of dust particles in coastal atmospheric environments. Overall, these findings provide new insights into the aging processes of mineral dust in coastal atmospheres and suggest potential interactions between dust particles and marine aerosols during heterogeneous halogen and sulfur processing in dust events.

## 1. Introduction

Atmospheric aerosols are complex mixtures of components originating from both natural and anthropogenic sources [1]. Among them, dust particles from dust storms represent one of the major natural aerosol sources, accounting for over half of the global aerosol burden [2] and significantly affecting air quality [3], human health [4,5], and climate dynamics [6]. These particles influence the Earth’s radiative budget by scattering and absorbing incoming solar radiation [6,7,8] and modulate cloud formation processes by acting as cloud condensation nuclei and ice nuclei [9,10]. Furthermore, long-range-transported dust can affect marine ecosystems by influencing phytoplankton productivity and oceanic CO_2_ uptake [11].

In East Asia, dust storms originate mainly from the Taklimakan and the Gobi Desert [3], which collectively account for 10–25% of global dust emissions [12]. These dust particles can traverse thousands of kilometers, reaching regions such as Japan, the Korea Peninsula, and the North Pacific [13,14,15]. During transport, dust undergoes chemical aging through interactions with pollutants such as SO_2_, NO_x_, and oxidants, leading to sulfation and nitration of mineral dust surfaces [16,17,18,19,20]. For example, during dust events in Shanghai, atmospheric oxidation of NO_x_ can promote the formation of HNO_3_, which subsequently reacts with NH_4_^+^ to form nitrate coatings on dust particles [21]. These nitrate layers not only modify the particles’ composition but also enhance their hygroscopicity, which in turn can facilitate the formation of secondary organic aerosols (SOAs) on the surface of dust particles [22].

Although the heterogeneous uptake of sulfur and nitrogen species on mineral dust has been extensively investigated, most previous studies have focused either on inland urban atmospheres dominated by anthropogenic pollutants or on remote marine atmospheres strongly influenced by sea-salt aerosols [23,24]. In coastal urban regions, however, mineral dust particles are simultaneously exposed to anthropogenic emissions and marine-derived species, potentially leading to distinct heterogeneous reaction pathways and particle aging processes. Previous observations in marine atmospheres have suggested that calcium-rich dust particles can undergo chloride enrichment and deliquescence through interactions with sea-salt aerosols [25]. Nevertheless, the chemical evolution of dust particles in complex coastal urban environments remains poorly understood, particularly regarding the combined effects of anthropogenic pollutants and marine aerosols on dust surface chemistry.

In this study, high-resolution scanning electron microscopy coupled with energy-dispersive X-ray spectroscopy (SEM-EDX) was employed to investigate the morphology and elemental composition of aerosol particles collected during four dust events in Qinhuangdao, a coastal city in Northern China. This study aims to elucidate the heterogeneous aging processes of mineral dust particles in coastal urban atmospheres and to evaluate the influences of anthropogenic pollutants and marine-derived species on dust surface chemistry at the single particle level.

## 2. Materials and Methods

### 2.1. Sampling Site and Sample Collection

**Figure 1:**
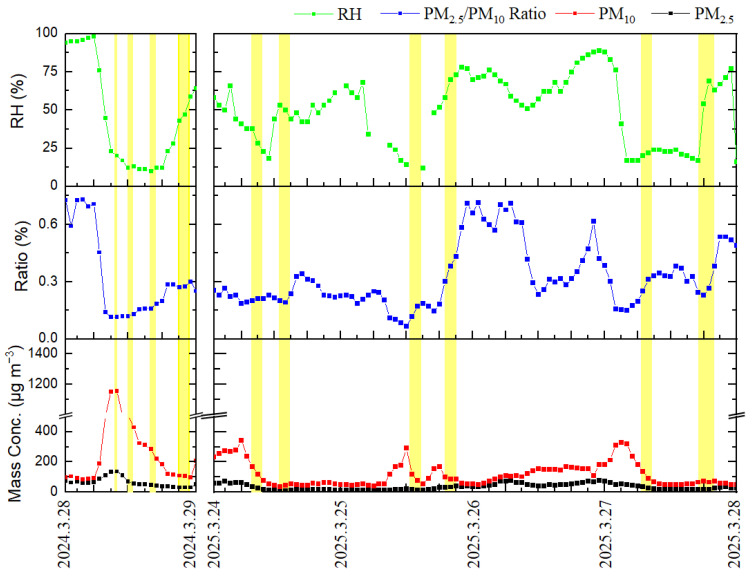
Temporal variations in PM_2.5_ and PM_10_ mass concentrations during dust events. The yellow bar showing the sampling durations.

Atmospheric dust samples were collected on the rooftop of a 15-story teaching building at the Qinhuangdao Campus of Northeastern University (119.58° E, 39.94° N). The site is situated approximately 1.5 km from the Bohai Sea coastline and 1.0 km from the West Ring Road, with minimal direct industrial influence and thus representative typical coastal urban atmospheric conditions (Figure S1). A minivol^TM^ portable cascade air sampler (Airmetrics Corp., Springfield, OR, USA) was operated at a flow rate of 5 L/min to collect particles onto polycarbonate filters (Merck Millipore Ltd., Cork, Ireland). Sampling durations ranged from 1 to 4 h, adjusted according to ambient particle loading. High particle loading under heavily polluted conditions may cause particle overlap on the filter membrane and interfere with single-particle analysis. Therefore, variable sampling durations were adopted to maintain appropriate particle number density on the filter membranes. Samples were collected throughout the entire duration of Dust Event A, from onset to dissipation, whereas only end-stage samples were collected for Dust Events B, C, and D. Detailed sample information is provided in Table 1 and Figure 1.

### 2.2. Sample Analysis

The collected samples were analyzed using a scanning electron microscope (SEM, FEI, Ltd., Hillsboro, OR, USA) equipped with an energy-dispersive X-ray spectrometer (EDX), according to the National Standard of the People’s Republic of China (GB/T 35099-2018) [26]. The procedure was as follows: a small piece of the polycarbonate membrane was mounted on a sample stub using conductive carbon adhesive tape. Subsequently, the samples were sputter-coated with a thin layer of platinum to enhance conductivity and improve image quality. Finally, the particles were observed at high magnification, and their elemental compositions were determined using EDX. SEM–EDX provides semi-quantitative information on the relative elemental abundances in individual particles. Elements with mass fractions greater than approximately 0.1 wt% can be detected using this EDX system.

### 2.3. Air Mass Backward Trajectory Analysis

To investigate the transport pathways and origins of air masses during the sampling periods, backward trajectories were calculated for each sampling event. Air mass backward trajectories were calculated using the NOAA HYSPLIT online model [27,28]. Trajectory endpoints were set at 500 m above ground level to characterize regional air mass transport within the lower boundary layer, which is closely associated with near-surface aerosol transport during dust events [18]. Although some uncertainty is inherent in trajectory analysis, the dominant transport pathways during the study period were generally consistent, supporting the reliability of the source attribution.

## 3. Results and Discussions

### 3.1. Characteristics of Dust Events

Figure 1 illustrates the temporal evolution of PM_2.5_ and PM_10_ mass concentrations along with relative humidity (RH) during four distinct dust events. The arrival of each dust event was characterized by distinct physicochemical signatures: (1) a sharp increase in PM_10_ mass concentrations within a few hours; (2) a dramatic reduction in RH within a few hours due to the influx of dry air masses originating from arid and semi-arid regions; and (3) a significant decrease in the PM_2.5_/PM_10_ ratio to below 0.3. These synchronous changes clearly demonstrate a rapid transition from fine-particle-dominated to coarse-particle-dominated conditions during dust events. The pronounced variations in both particulate matter characteristics and meteorological parameters underscore the substantial impact of mineral dust transport on downwind air quality. Backward trajectories (Figure S2) further demonstrated that the dust originated mainly from Mongolia and Inner Mongolia in northwestern China.

### 3.2. Single-Particle Classification of Dust Particles

**Figure 2:**
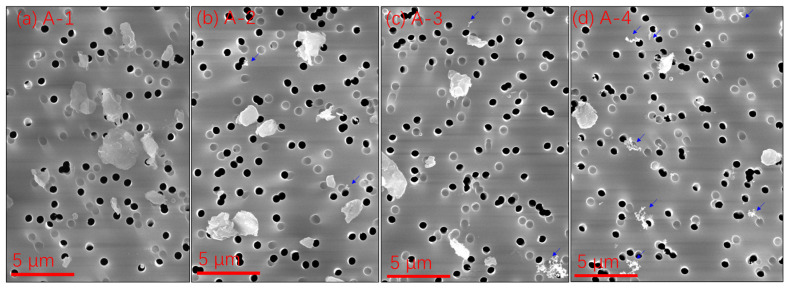
Low-magnification SEM micrographs of aerosol particles collected during Dust Event A. (**a**–**d**) represents the dust samples collected from the initial stages to the end stages. The images show that mineral dust was the dominant particle type during the dust event. Anthropogenic particles, particularly soot particles (some of which are indicated by blue arrows), gradually increased as the dust event progressed. The sampling durations for samples A-1, A-2, A-3, and A-4 were 1, 2, 2, and 3 h, respectively.

Single particles can be broadly classified into two categories based on their dominant elemental compositions: (1) carbonaceous particles, including organic and soot particles, primarily composed of carbon and oxygen; (2) non-carbonaceous particles, characterized by complex elemental compositions (e.g., Si, Al, Fe, Ca, K, Na, Cl) [1,29]. Soot particles are widely recognized as reliable tracers of anthropogenic combustion emissions [30,31,32,33]. As shown in Figure 2, the fraction of soot particles during dust events was relatively low compared with that observed on non-dust days in a previous study [34]. However, the gradual increase in soot particle fractions as the dust events progressed may reflect enhanced interactions between transported dust particles and local anthropogenic emissions.

**Figure 3:**
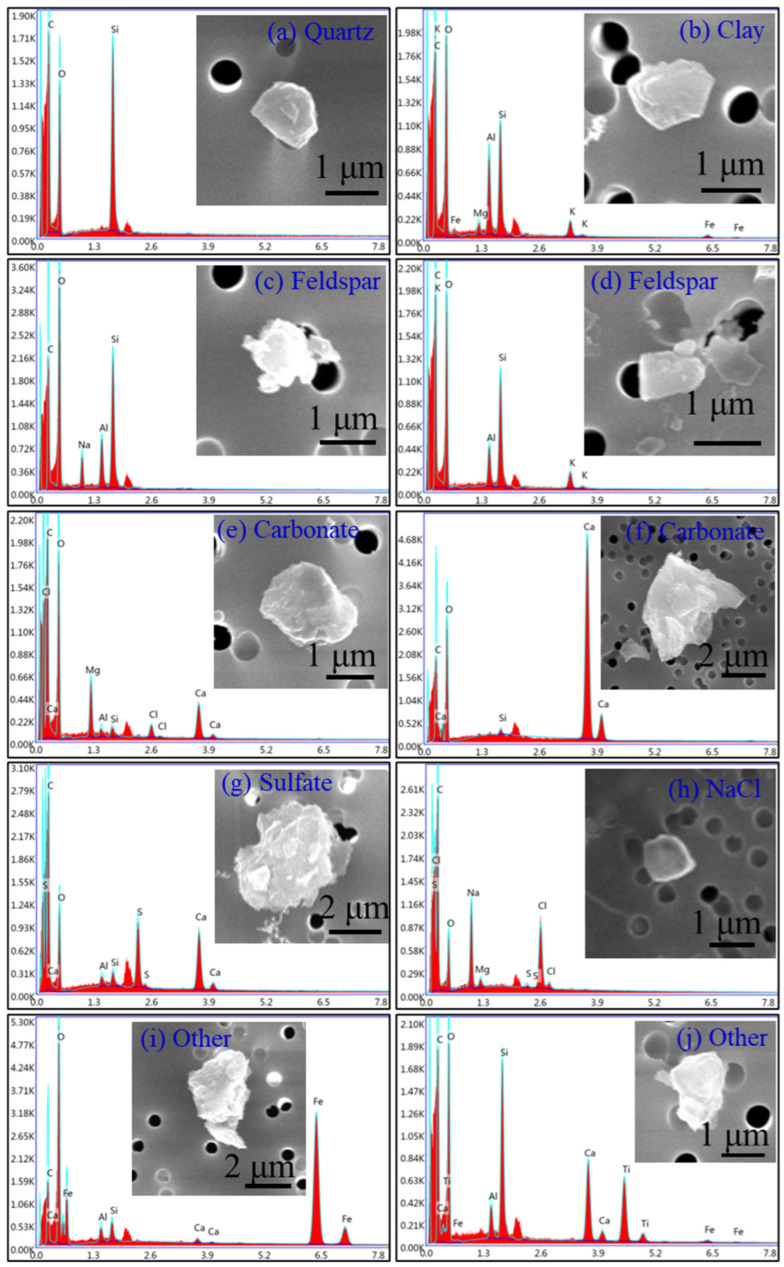
Morphologies and elemental compositions of single non-carbonaceous particles. (**a**) Quartz particle; (**b**) Clay mineral particle; (**c**,**d**) Feldspar particles (plagioclase and potassium feldspar); (**e**,**f**) Carbonate mineral particles (dolomite and calcite); (**g**) Sulfate particle; (**h**) NaCl particle; (**i**) Iron oxide particle; (**j**) Complex particle. Panels (**i**,**j**) were classified as “other” particle types.

To investigate the role of mineral dust in atmospheric heterogeneous chemistry, subsequent analyses focused primarily on non-carbonaceous particles. In total, 449 particles from Dust Event A (109 for A-1, 131 for A-2, 111 for A-3, and 98 for A-4), 234 particles from Dust Event B (125 for B-1 and 109 for B-2), 216 particles from Dust Event C (105 for C-1 and 111 for C-2), and 231 particles from Dust Event D (118 for D-1 and 113 for D-2) were analyzed. According to the main elemental composition of single particles [18,23], they were subsequently classified into the following subtypes based on their elemental characteristics: quartz, clay minerals, carbonate, sulfate, NaCl, and other types, as shown in Figure 3.

Quartz particles were mainly composed of Si and O, and were readily identified by their high SiO_2_ composition, as shown in Figure 3a. Si(Al)-rich particles predominantly consist of aluminosilicate minerals, especially clay minerals (Figure 3b) and feldspars (Figure 3c,d). These mineral groups exhibit distinct Si/Al weight ratios: clay minerals typically display ratios of 1–2, while feldspars show a higher characteristic ratio of approximately 3 [18,23]. Feldspar particles were further classified into plagioclase (Figure 3c), characterized by the presence of Na, and potassium feldspar (Figure 3d), characterized by K. Carbonate particles mainly consisted of calcium- and magnesium-containing carbonates, including dolomite (Figure 3f; CaCO_3_) and dolomite (Figure 3e; CaMg(CO_3_)_2_). NaCl particles were mainly composed of Na and Cl (Figure 3h). Additional particle types observed in the samples included various metal oxides and mixed aluminosilicate-carbonate particles.

**Figure 4:**
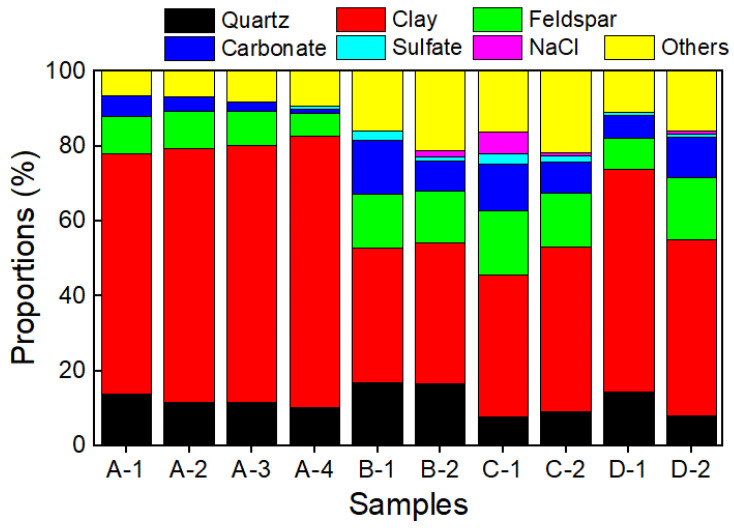
Relative abundances of different particle types during the dust events. The particle-type distributions exhibited relatively consistent patterns among the different dust events. Overall, the particle population was dominated by clay minerals, quartz, feldspar, and carbonate particles.

As shown in Figure 4, the mineralogical composition during the four dust storms was consistently dominated by clay minerals, which accounted for an average of 53.5% (ranging from 36.0% to 72.4%). These were followed by feldspar (12.0% on average; range: 6.1–17.1%) and quartz (11.9% on average; range: 7.6–16.8%). Carbonate particles accounted for an average of 7.3% (2.7–14.4%), while only minor fractions of NaCl and sulfate particles were present, with average fractions of 0.9% and 1.1%, respectively. This particle assemblage, characteristic of Asian dust, is consistent with the backward trajectory analysis (Figure S2), which indicated that the air masses mainly originated from the Gobi Desert in Mongolia and Inner Mongolia, China. Nonetheless, the observed variations in the abundances of these particle types may be attributed to spatial heterogeneity in the geochemical composition of the source regions [35,36].

### 3.3. Sulfur Enrichment on Mineral Dust Surfaces

During Dust Event A, the proportion of sulfur (S)-containing particles rose significantly, from 16.5% initially to 41.8% by the end of the event. Likewise, elevated proportions of S-containing particles were observed at the end of Dust Events B, C, and D, reaching 44.0%, 80.2%, and 45.1%, respectively. These results suggest that heterogeneous reactions on mineral dust surfaces were limited during the initial stages of the dust events. This may be because the dust plumes were relatively cold and dry (Figure 1), with limited mixing with local warm and humid polluted air masses, conditions that are unfavorable for heterogeneous reactions on dust surfaces [37,38]. However, sulfate formation on dust surfaces appeared to increase continuously as the dust events progressed. This trend may be attributed to the gradual mixing of dust plumes with local polluted air masses, as evidenced by the increasing abundance of anthropogenic particles (soot; Figure 2) and the gradual increase in RH (Figure 1).

Although only Dust Event A was sampled throughout the entire event, whereas Dust Events B, C, and D were sampled only during the later stages, our observations are generally consistent with previous studies showing that heterogeneous reactions on mineral dust particles are initially limited during dust transport [18,37]. Our findings collectively suggest that sulfate formation on dust surfaces is mainly influenced by two key factors: the transport history of dust plumes over polluted regions and the extent of mixing with polluted air masses [19,37,38,39].

### 3.4. Chlorine Enrichment on Mineral Dust Surfaces

**Figure 5:**
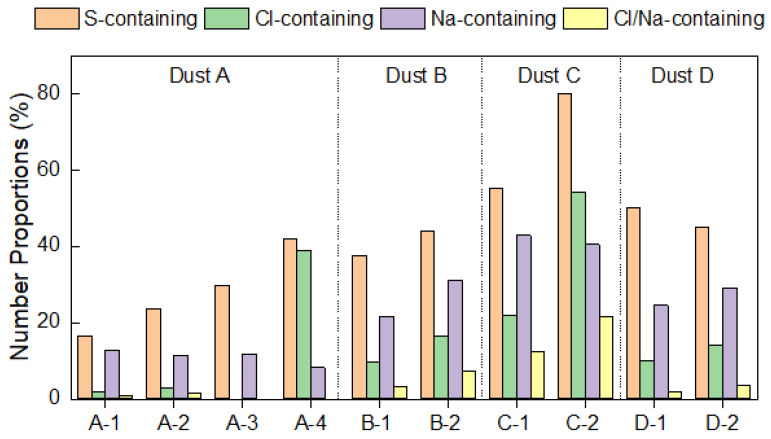
Relative abundances of sulfur (S)-containing and chlorine (Cl)-containing particles during the four dust events. The abundance of S-containing particles increased as the dust events progressed, suggesting enhanced sulfate formation on mineral dust particles during transport. Elevated abundances of Cl-containing particles during the later stages of the dust events suggest enhanced chlorine enrichment on mineral dust particles in coastal urban environments.

Single-particle analysis showed a gradual increase in chlorine (Cl)-containing particles as the dust events progressed, a trend similar to that observed for sulfur-containing particles (Figure 5). For instance, Cl-containing particles accounted for only 2.4% of all analyzed particles during the initial stage of Dust Event A but increased significantly to 38.8% by the end of the event. Similarly high proportions of Cl-containing particles were observed during the later stages of Dust Events B, C, and D (Figure 5). Given the low abundance of Cl-containing particles during the initial stages in this study and in previous research [40], it is unlikely that chlorine was primarily derived from dust sources. These results suggest that substantial chlorine enrichment likely occurred on mineral dust surfaces during dust transport and aging processes.

To better understand the potential sources and processes responsible for chlorine enrichment on mineral dust particles, we compared our findings with previous studies conducted in inland cities [38]. In contrast to the coastal urban environment studied here, those investigations reported only minor proportions of Cl-containing particles throughout dust events in inland regions. These observations suggest that marine-derived aerosols, particularly sea-spray aerosols, may contribute to chlorine enrichment on mineral dust particles in coastal atmospheres.

Sea-spray aerosols are rich in NaCl, which can undergo significant chemical transformations during atmospheric aging processes. One important transformation pathway involves chloride depletion through acid displacement reactions with acidic species such as H_2_SO_4_ and HNO_3_, resulting in the release of gaseous chlorine species, particularly HCl [24,41,42,43]. The released HCl may subsequently be taken up by mineral dust particles through heterogeneous processes [25]. In addition, dust particles may also acquire chlorine through physical mixing or coagulation with aged sea-salt particles [44]. Therefore, both heterogeneous uptake of gaseous chlorine species and particle mixing processes may contribute to the observed chlorine enrichment on mineral dust particles.

**Figure 6:**
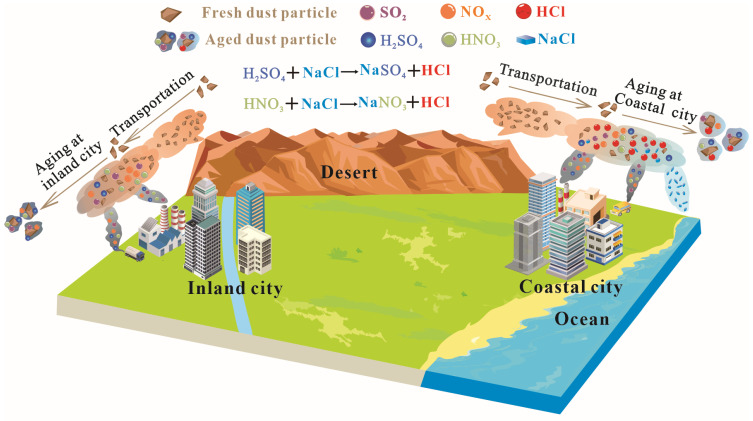
A diagram showing the possible heterogeneous reactions of mineral dust particles in inland cities and coastal cities.

In this study, however, only a small fraction of intact NaCl particles were identified (Figure 4). Moreover, although the abundance of Na-containing particles did not increase significantly during the dust events, Cl-containing particles were substantially more abundant than particles containing both Cl and Na simultaneously. These observations suggest that most sea-salt particles had undergone substantial heterogeneous aging prior to sampling. The results further suggest that heterogeneous uptake of gaseous HCl may have contributed to the observed chlorine enrichment on mineral dust particles, as illustrated in Figure 6.

Nevertheless, it should be noted that sea-salt aerosols can undergo complex atmospheric aging processes and produce a variety of reactive chlorine-containing species, including HCl, Cl_2_, and ClNO_2_ [42]. These reactive chlorine species may also participate in heterogeneous reactions on mineral dust surfaces. Therefore, the observed chlorine enrichment on dust particles cannot be attributed exclusively to HCl uptake. Furthermore, SEM–EDX analysis mainly provides elemental information and has limited sensitivity for nitrogen detection, making it difficult to directly identify specific chemical species or molecular speciation. Consequently, the detailed pathways and chemical speciation associated with chlorine enrichment on dust particles remain uncertain and require further investigation using complementary analytical techniques.

### 3.5. Atmospheric Implications and Limitations

It is well established that marine aerosols are rich in reactive chlorine species and can interact with long-range-transported mineral dust. Previous studies have documented substantial chlorine enrichment on dust particles, particularly in remote marine environments and marine boundary layers [14,15,24,25]. For example, Sullivan et al. [24] observed that approximately 65% of dust particles in the marine boundary layer contained chlorine, suggesting substantial chlorine processing during transport over oceanic regions. Our previous study also revealed pronounced chlorine enrichment on dust particles after marine transport, with 40.7–76.3% of dust particles containing chlorine after the air masses had passed over marine regions before reaching the coastal site in Qinhuangdao [40].

In the present study, however, backward trajectory analysis indicated that the dust plumes were transported directly to the sampling site without prolonged transport through the marine boundary layer (Figure S2). Despite the absence of prolonged marine transport, substantial proportions of Cl-containing dust particles were still observed during the later stages of the dust events. This finding suggests that mineral dust particles in coastal urban environments may become enriched in chlorine through interactions with locally emitted marine-derived chlorine species, potentially facilitated by anthropogenic pollutants and concurrent sulfate formation processes (Figure 6). Therefore, coastal urban environments may represent unique atmospheric settings in which marine and anthropogenic influences jointly modify the surface chemistry of transported dust particles.

Nevertheless, the chlorine enrichment observed in this study appeared less extensive than that reported in remote marine environments. Although a considerable fraction of particles contained chlorine, the average Cl weight percentage on single particles was relatively low (4.02%), suggesting that chlorine enrichment mainly occurred as a surface-level modification rather than bulk incorporation within the particles. Previous studies conducted in coastal cities have generally focused on bulk aerosol chemical composition, in which the relative contribution of chlorine was often minor and therefore received limited attention [19]. In contrast, the SEM–EDX single-particle analysis used in this study enabled the detection of subtle elemental changes on individual dust particles, providing evidence of chlorine enrichment during dust aging in coastal urban atmospheres.

Chlorine enrichment can substantially alter the physicochemical properties of mineral dust particles, particularly their hygroscopicity and heterogeneous reactivity. Tobo et al. [25] demonstrated that chlorinated Asian dust particles could transform into aqueous droplets during long-range transport. Although the extent of chlorine enrichment observed in this study was relatively moderate, such surface modification may still influence the hygroscopic behavior and atmospheric aging of dust particles. Previous studies have suggested that chlorine-containing dust particles may facilitate the formation of reactive chlorine species such as ClNO_2_ through heterogeneous reactions, even under relatively low RH conditions [45,46]. Since ClNO_2_ is an important nighttime chlorine reservoir and photolabile precursor, its formation may further influence atmospheric oxidation capacity and secondary aerosol production in coastal urban regions.

Furthermore, although mineral dust is commonly transported from inland source regions toward marine environments, our trajectory analysis suggests that some dust-laden air masses may subsequently recirculate inland after passing through coastal regions (Figure S3). Such recirculation processes could redistribute chemically aged dust particles enriched with sulfate and chlorine back to inland urban areas, thereby further modifying the physicochemical evolution and environmental impacts of transported aerosols during regional transport.

## 4. Conclusions

In this study, single dust particles collected during four dust events in the coastal city of Qinhuangdao were analyzed using high-resolution scanning electron microscopy coupled with an energy-dispersive X-ray spectrometer (SEM–EDX). The results indicated that the dust was predominantly composed of clay minerals (53.5% ± 13.7%), feldspar (12.0% ± 3.6%), quartz (11.9% ± 3.2%), and carbonate particles (7.3% ± 4.1%), whereas sulfate and NaCl particles accounted for only minor fractions. Elemental analysis revealed a progressive increase in sulfur (S)-containing particles as the dust events progressed, suggesting continuous sulfate formation on mineral dust surfaces. More importantly, a substantial increase in chlorine-containing particles (excluding NaCl) was observed, providing evidence that chlorine enrichment can occur in coastal urban atmospheres even without prolonged transport through remote marine boundary layers.

These findings suggest that coastal urban regions represent unique environments for dust aging, where marine-derived chlorine species and anthropogenic pollutants can jointly modify the surface chemistry of mineral dust particles. Compared with inland cities, the coexistence of marine aerosols and anthropogenic emissions in coastal atmospheres may promote more complex heterogeneous reactions, leading to distinct physicochemical evolution of transported dust particles. Although the observed chlorine enrichment was relatively limited compared with that reported in remote marine environments, even moderate sulfate and chlorine enrichment may alter the hygroscopicity and heterogeneous reactivity of mineral dust, potentially influencing secondary aerosol formation and reactive chlorine chemistry. Such aging processes may further influence the atmospheric behavior of transported dust aerosols in downwind regions. This study highlights the important role of coupled marine and anthropogenic influences in shaping the aging processes of mineral dust in coastal urban atmospheres.

## 5. Future Perspectives

This study suggests that mineral dust particles in coastal urban atmospheres can undergo chlorine enrichment through interactions with marine-derived chlorine species and anthropogenic pollutants. However, the detailed reaction mechanisms, chemical speciation, and atmospheric implications associated with chlorine enrichment on dust particles remain insufficiently understood. Therefore, future studies should: (1) combine single-particle microscopic analysis with bulk chemical composition measurements and gaseous pollutant observations to comprehensively characterize particle morphology, bulk chemical composition, and precursor gases, thereby helping to identify the major processes contributing to chlorine enrichment on dust surfaces; (2) apply complementary analytical techniques capable of resolving molecular-level composition and nitrogen-containing species to better identify the specific chlorine-containing products formed during atmospheric aging; and (3) conduct further laboratory and field investigations to evaluate the impacts of chlorine enrichment on the physicochemical properties of dust particles. Hygroscopicity measurements, heterogeneous aging experiments, and cloud activation studies would help determine how chlorine enrichment influences water uptake, particle reactivity, and secondary aerosol formation.

## Figures and Tables

**Table 1:** Sample information during dust events. RH is relative humidity.

Sample ID	Sampling Time	PM_2.5_	PM_10_	PM_2.5_/PM_10_	RH
	(UTC + 8)	(μg m^−3^)	(μg m^−3^)		%
Dust A-1	28 March 2024 9:00–10:00	134	1152	0.12	20
Dust A-2	28 March 2024 11:00–13:00	63	506	0.13	13
Dust A-3	28 March 2024 15:00–17:00	43	252	0.17	11
Dust A-4	28 March 2024 20:00–23:00	29	103	0.28	50
Dust B-1	24 March 2025 7:00–10:00	25	121	0.21	29
Dust B-2	24 March 2025 12:00–15:00	9	43	0.21	49
Dust C-1	25 March 2025 12:00–15:00	12	83	0.16	12
Dust C-2	25 March 2025 18:00–21:00	33	89	0.37	67
Dust D-1	27 March 2025 7:00–10:00	23	69	0.33	23
Dust D-2	27 March 2025 16:00–20:00	19	68	0.28	51

## Data Availability

Data is contained within the article and the Supplementary Materials.

## Supplementary Materials

The following supporting information can be downloaded at https://www.mdpi.com/article/10.3390/toxics14060460/s1: Figure S1: Map showing the geographical locations of Qinhuangdao City. Figure S2: 48 h air mass backward trajectories during dust periods at the sampling site in Qinhuangdao city. Figure S3: 48 h air mass forward trajectories during dust periods at the sampling site in Qinhuangdao city.

## References

[1] Shao L., Liu P., Jones T., Yang S., Wang W., Zhang D., Li Y., Yang C.-X., Xing J., Hou C. (2022). A review of atmospheric individual particle analyses: Methodologies and applications in environmental research. Gondwana Res..

[2] Bibi M., Saad M., Masmoudi M., Laurent B., Alfaro S.C. (2020). Long-term (1980–2018) spatial and temporal variability of the atmospheric dust load and deposition fluxes along the North-African coast of the Mediterranean Sea. Atmos. Res..

[3] Chen S., Zhao D., Huang J., He J., Chen Y., Chen J., Bi H., Lou G., Du S., Zhang Y. (2023). Mongolia Contributed More than 42% of the Dust Concentrations in Northern China in March and April 2023. Adv. Atmos. Sci..

[4] Qin L., Yang L., Liu L., Tong S., Liu Q., Li G., Zhang H., Zhu W., Liu G., Zheng M. (2024). Oxidative potential and persistent free radicals in dust storm particles and their associations with hospitalization. Nat. Commun..

[5] Schiavo B., Meza-Figueroa D., Vizuete-Jaramillo E., Robles-Morua A., Angulo-Molina A., Reyes-Castro P.A., Inguaggiato C., Gonzalez-Grijalva B., Pedroza-Montero M. (2023). Oxidative potential of metal-polluted urban dust as a potential environmental stressor for chronic diseases. Environ. Geochem. Health.

[6] Kok J.F., Storelvmo T., Karydis V.A., Adebiyi A.A., Mahowald N.M., Evan A.T., He C., Leung D.M. (2023). Mineral dust aerosol impacts on global climate and climate change. Nat. Rev. Earth Environ..

[7] Adebiyi A., Kok J.F., Murray B.J., Ryder C.L., Stuut J.-B.W., Kahn R.A., Knippertz P., Formenti P., Mahowald N.M., Pérez García-Pando C. (2023). A review of coarse mineral dust in the Earth system. Aeolian Res..

[8] Devi M., Mishra S.K., Jayakumar A., Kaur S., Goel V., Narayanasamy V., Basheed G.A., Pandey K. (2025). Physico-chemical characterization of atmospheric particles during two intense dust storms in the vicinity of Thar Desert. Atmos. Pollut. Res..

[9] Kok J.F., Ward D.S., Mahowald N.M., Evan A.T. (2018). Global and regional importance of the direct dust-climate feedback. Nat. Commun..

[10] Yin Z., Yi F., He Y., Liu F., Yu C., Zhang Y., Wang W. (2021). Asian dust impacts on heterogeneous ice formation at Wuhan based on polarization lidar measurements. Atmos. Environ..

[11] Wang F., Zhao X., Gerlein-Safdi C., Mu Y., Wang D., Lu Q. (2017). Global sources, emissions, transport and deposition of dust and sand and their effects on the climate and environment: A review. Front. Environ. Sci. Eng..

[12] Hu Z., Huang J., Zhao C., Bi J., Jin Q., Qian Y., Leung L.R., Feng T., Chen S., Ma J. (2019). Modeling the contributions of Northern Hemisphere dust sources to dust outflow from East Asia. Atmos. Environ..

[13] Zhang T., Zheng M., Sun X., Chen H., Wang Y., Fan X., Pan Y., Quan J., Liu J., Wang Y. (2023). Environmental impacts of three Asian dust events in the northern China and the northwestern Pacific in spring 2021. Sci. Total Environ..

[14] Zhang D., Iwasaka Y. (2001). Chlorine deposition on dust particles in marine atmosphere. Geophys. Res. Lett..

[15] Tobo Y., Zhang D., Nakata N., Yamada M., Ogata H., Hara K., Iwasaka Y. (2009). Hygroscopic mineral dust particles as influenced by chlorine chemistry in the marine atmosphere. Geophys. Res. Lett..

[16] Wang Z., Pan X.L., Uno I., Chen X.S., Yamamoto S., Zheng H.T., Li J., Wang Z.F. (2018). Importance of mineral dust and anthropogenic pollutants mixing during a long-lasting high PM event over East Asia. Environ. Pollut..

[17] Tang M., Jia X., Chen L., Gu W., Huang C., Wang F., Luo L., Wang H., Wang X., Peng C. (2023). Heterogeneous reaction of NO_2_ with feldspar, three clay minerals and Arizona Test Dust. J. Environ. Sci..

[18] Wang W., Zhou H., Gao Y., Lyu R., Xing J., Zhou X., Li X., Shao L. (2024). Morphology and chemical composition of mineral particles in a special dust storm with high relative humidity in North China. Environ. Technol. Innov..

[19] Sun N., Wu L., Zheng F., Liang D., Qi F., Song S., Peng J., Zhang Y., Mao H. (2024). Atmospheric environment characteristic of severe dust storms and its impact on sulfate formation in downstream city. Sci. Total Environ..

[20] Huang L., Zhao Y., Li H., Chen Z. (2015). Kinetics of Heterogeneous Reaction of Sulfur Dioxide on Authentic Mineral Dust: Effects of Relative Humidity and Hydrogen Peroxide. Environ. Sci. Technol..

[21] Wu C., Zhang S., Wang G., Lv S., Li D., Liu L., Li J., Liu S., Du W., Meng J. (2020). Efficient Heterogeneous Formation of Ammonium Nitrate on the Saline Mineral Particle Surface in the Atmosphere of East Asia during Dust Storm Periods. Environ. Sci. Technol..

[22] Li W., Ito A., Wang G., Zhi M., Xu L., Yuan Q., Zhang J., Liu L., Wu F., Laskin A. (2025). Aqueous-phase secondary organic aerosol formation on mineral dust. Natl. Sci. Rev..

[23] Li J., Shao L.Y., Chang L.L., Xing J.P., Wang W.H., Li W.J., Zhang D.Z. (2018). Physicochemical Characteristics and Possible Sources of Individual Mineral Particles in a Dust Storm Episode in Beijing, China. Atmosphere.

[24] Sullivan R.C., Guazzotti S.A., Sodeman D.A., Tang Y., Carmichael G.R., Prather K.A. (2007). Mineral dust is a sink for chlorine in the marine boundary layer. Atmos. Environ..

[25] Tobo Y., Zhang D., Matsuki A., Iwasaka Y. (2010). Asian dust particles converted into aqueous droplets under remote marine atmospheric conditions. Proc. Natl. Acad. Sci. USA.

[26] (2018). Microbeam Analysis—Scanning Electron Microscopy with Energy Dispersive X-Ray Spectrome-Try—Morphology and Element Analysis of Single Fine Particles in Ambient Air.

[27] Stein A.F., Draxler R.R., Rolph G.D., Stunder B.J.B., Cohen M.D., Ngan F. (2015). NOAA’s HYSPLIT Atmospheric Transport and Dispersion Modeling System. Bull. Am. Meteorol. Soc..

[28] Rolph G., Stein A., Stunder B. (2017). Real-time Environmental Applications and Display sYstem: READY. Environ. Model. Softw..

[29] Niu H., Wu C., Ma X., Ji X., Tian Y., Wang J. (2024). Evaluation of single particle morphological characteristics and human health risks in different functional areas. World J. Eng..

[30] Bhandari J., China S., Chandrakar K.K., Kinney G., Cantrell W., Shaw R.A., Mazzoleni L.R., Girotto G., Sharma N., Gorkowski K. (2019). Extensive Soot Compaction by Cloud Processing from Laboratory and Field Observations. Sci. Rep..

[31] Pang Y., Wang Y., Wang Z., Zhang Y., Liu L., Kong S., Liu F., Shi Z., Li W. (2022). Quantifying the Fractal Dimension and Morphology of Individual Atmospheric Soot Aggregates. J. Geophys. Res. Atmos..

[32] Berrellez-Reyes F., Schiavo B., Gonzalez-Grijalva B., Angulo-Molina A., Meza-Figueroa D. (2025). Characterization of soot and crystalline atmospheric ultrafine particles. Environ. Pollut..

[33] Niu H., Wu C., Schindler M., Silva L.F.O., Ma B., Ma X., Ji X., Tian Y., Zhu H., Bao X. (2024). Characterization of PM_2.5_ Carbonaceous Components in a Typical Industrial City in China under Continuous Mitigation Measures. Toxics.

[34] Wang W., Wang M., Shao L., Zhou X., Zhao Z., Li N., Zhou H., Li W. (2024). Morphology and elemental composition of individual solid dust particles: From different sources to the atmosphere. Atmos. Environ..

[35] Zhao W., Liu L., Chen J., Ji J. (2019). Geochemical characterization of major elements in desert sediments and implications for the Chinese loess source. Sci. China Earth Sci..

[36] Wei G., Zhang C., Li Q., Wang H., Wang R., Zhang Y., Yuan Y. (2023). Characterization of geochemical elements in surface sediments from Chinese deserts. CATENA.

[37] Wu F., Zhang D.Z., Cao J.J., Guo X., Xia Y., Zhang T., Lu H., Cheng Y. (2017). Limited production of sulfate and nitrate on front-associated dust storm particles moving from desert to distant populated areas in northwestern China. Atmos. Chem. Phys..

[38] Wang Z., Hu W., Niu H., Hu W., Wu Y., Wu L., Ren L., Deng J., Guo S., Wu Z. (2021). Variations in physicochemical properties of airborne particles during a heavy haze-to-dust episode in Beijing. Sci. Total Environ..

[39] Song L., Bi X., Zhang Z., Li L., Dai Q., Zhang W., Li H., Wang X., Liang D., Feng Y. (2022). Impact of sand and dust storms on the atmospheric environment and its source in Tianjin-China. Sci. Total Environ..

[40] Wang W., Zhou H., Lyu R., Li W., Zhao Z., Zhou X., Shao L. (2025). Chemical evaluation of aerosol particles in an intense Asian dust storm in a coastal city: Direct vs. reverse transport stages. J. Environ. Sci..

[41] Sarin M., Kumar A., Srinivas B., Sudheer A.K., Rastogi N. (2010). Anthropogenic sulphate aerosols and large Cl-deficit in marine atmospheric boundary layer of tropical Bay of Bengal. J. Atmos. Chem..

[42] Su B., Wang T., Zhang G., Liang Y., Lv C., Hu Y., Li L., Zhou Z., Wang X., Bi X. (2022). A review of atmospheric aging of sea spray aerosols: Potential factors affecting chloride depletion. Atmos. Environ..

[43] Xu L., Liu X., Gao H., Yao X., Zhang D., Bi L., Liu L., Zhang J., Zhang Y., Wang Y. (2021). Long-range transport of anthropogenic air pollutants into the marine air: Insight into fine particle transport and chloride depletion on sea salts. Atmos. Chem. Phys..

[44] Zhang L., Wang Y., Xie W., Li W., Kojima T., Zhang D. (2024). High heterogeneity and aging state of mineral particles in a slowly-moving dust plume on the southwestern coast of Japan. Sci. Total Environ..

[45] Wang H., Peng C., Wang X., Lou S., Lu K., Gan G., Jia X., Chen X., Chen J., Wang H. (2022). N_2_O_5_ uptake onto saline mineral dust: A potential missing source of tropospheric ClNO_2_ in inland China. Atmos. Chem. Phys..

[46] Royer H.M., Mitroo D., Hayes S.M., Haas S.M., Pratt K.A., Blackwelder P.L., Gill T.E., Gaston C.J. (2021). The Role of Hydrates, Competing Chemical Constituents, and Surface Composition on ClNO_2_ Formation. Environ. Sci. Technol..

