# Supplementary material for: Heterogeneous Chlorine Reactions on Mineral Dust During Dust Storm Events in the Coastal City of Qinhuangdao
Source: Toxics. 2026 May 25;14(6):460. doi: 10.3390/toxics14060460 (PMC13306625; doi:10.3390/toxics14060460)
Supplement: Supplementary file 1 [file toxics-14-00460-s001.zip › toxics-4300199-supplementary.pdf]

Supporting information for "Heterogeneous Chlorine Reactions on Mineral Dust During Dust Storm Events in the Coastal City of Qinhuangdao"

Yulong Wang[1,2], Jiajia Shao[1,2], Ting Wei[1,2], Ruihe Lyu[3], Pengju Liu[4], Chen Lin[2], Wenhua Wang[1,2*], Longyi Shao[5]

1. School of Resources and Civil Engineering, Northeastern University, Shenyang 110819, China.
2. School of Resources and Materials, Northeastern University at Qinhuangdao, Qinhuangdao 066004, China.
3. College of Marine Resources and Environment, Hebei Normal University of Science & Technology, Qinhuangdao 066004, China.
4. State Key Laboratory of Regional Environment and Sustainability, School of Environment, Tsinghua University, Beijing, 100084, China.
5. College of Geosciences and Surveying Engineering, China University of Mining and Technology, Beijing, 100083, China.

Corresponding author: (wangwenhua@neuq.edu.cn)

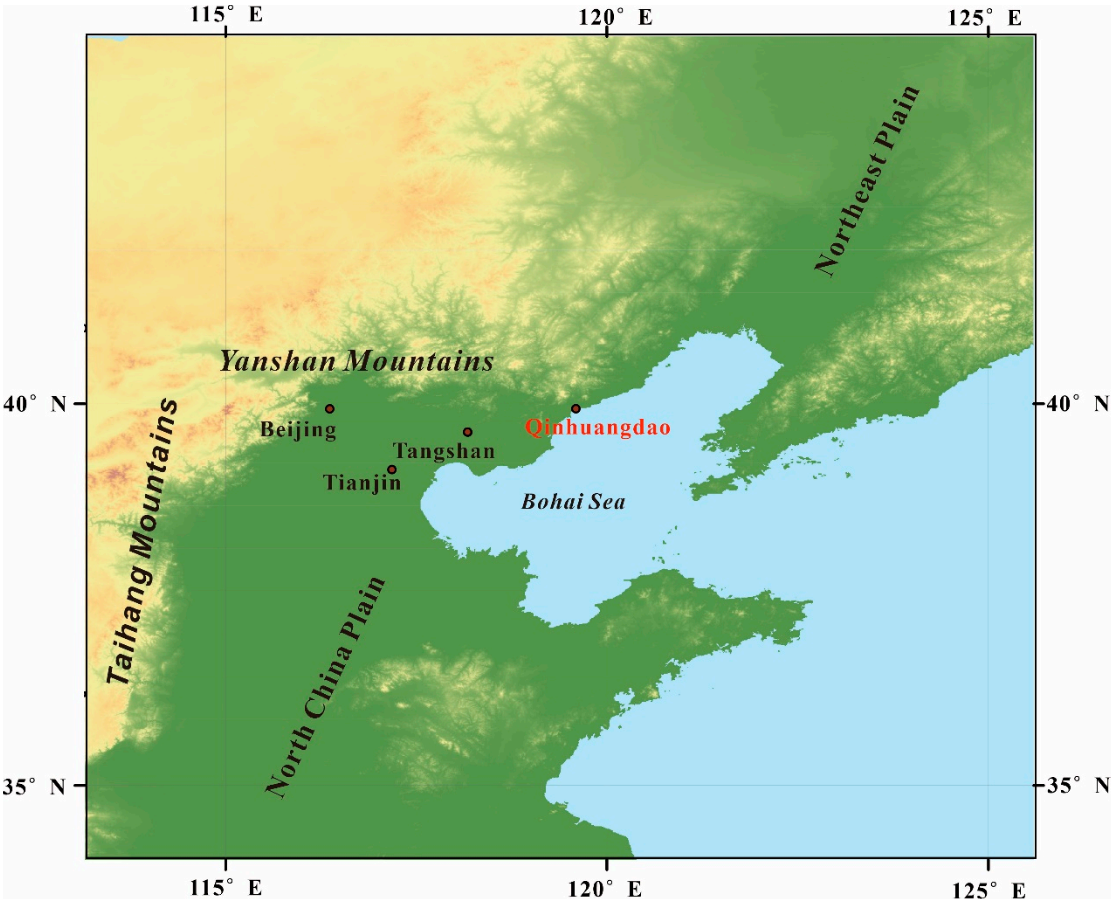

Figure S1 Map showing the geographical locations of Qinhuangdao City.

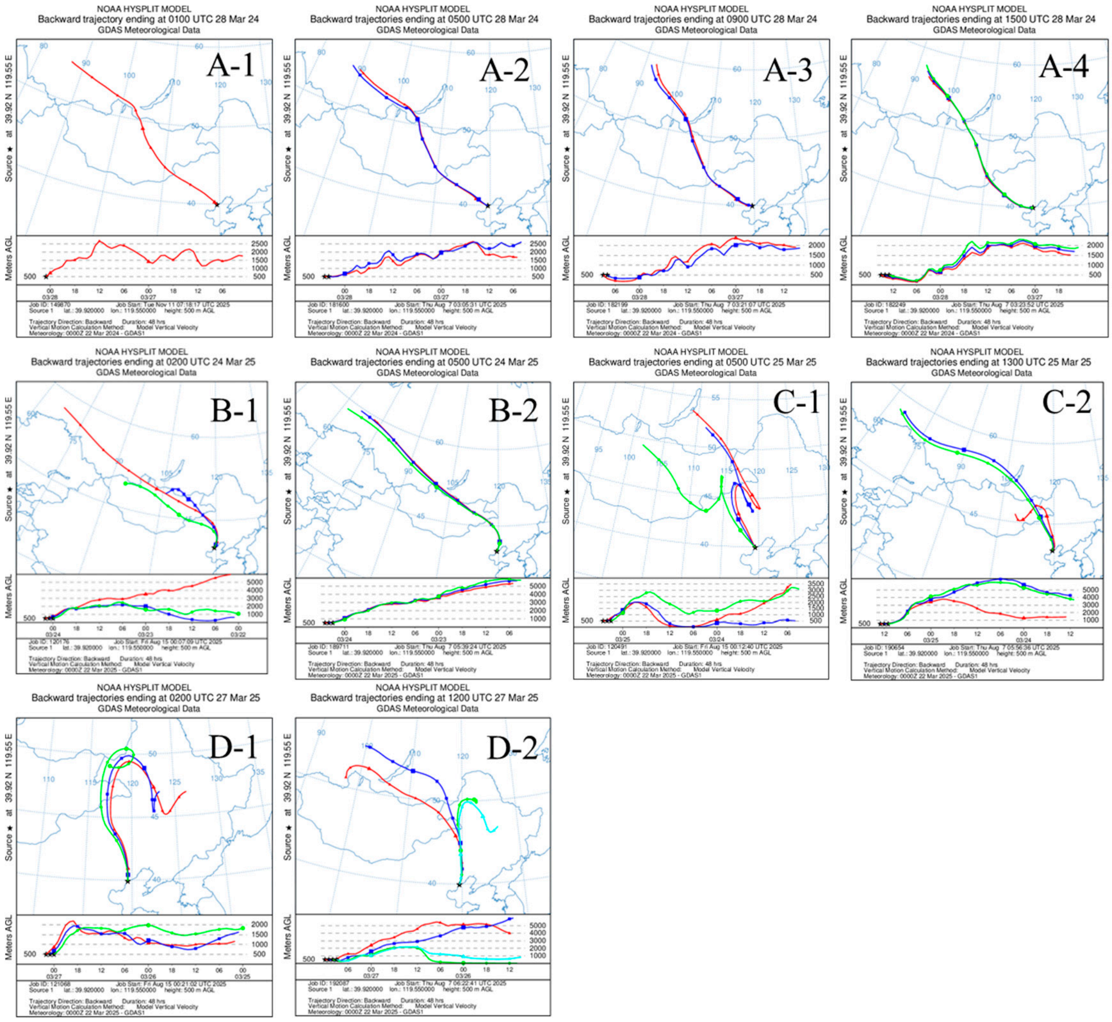

Figure S2 48-h air mass backward trajectories during dust periods at the sampling site in Qinhuangdao city. (https://ready.arl.noaa.gov/HYSPLIT_traj.php)

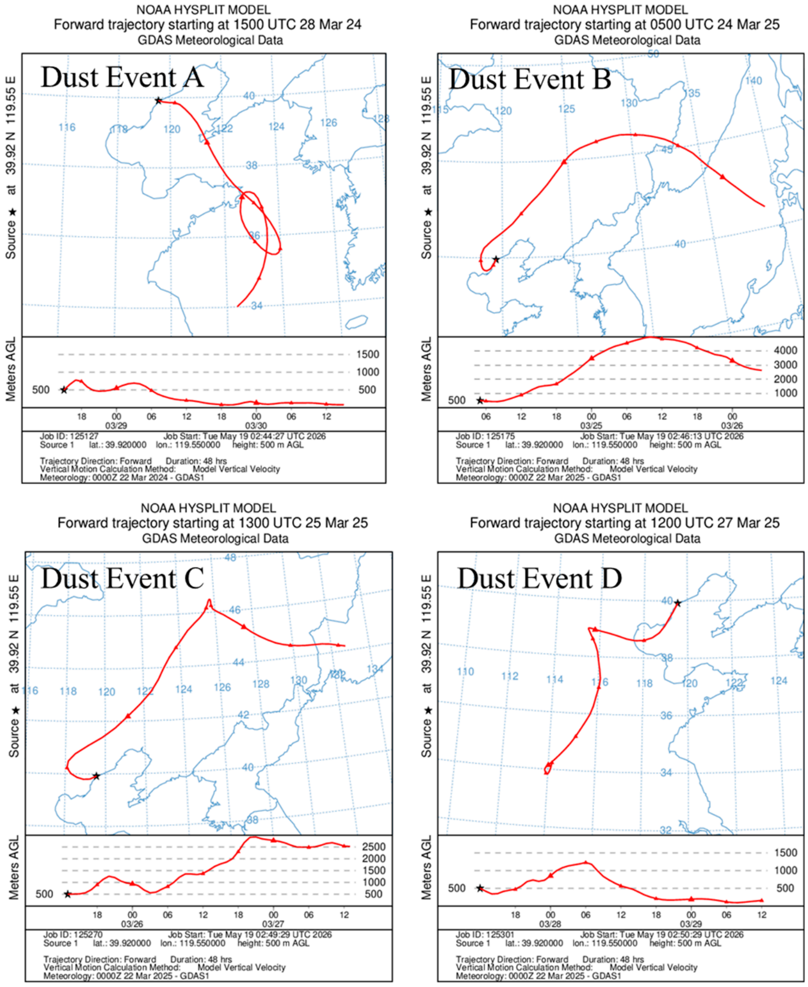

Figure S3 48-h air mass forward trajectories during dust periods at the sampling site in Qinhuangdao city. (https://ready.arl.noaa.gov/HYSPLIT_traj.php)
